# Alzheimer’s Disease, Neuroprotection, and CNS Immunosenescence

**DOI:** 10.3389/fphar.2012.00138

**Published:** 2012-07-17

**Authors:** Wolfgang J. Streit, Qing-Shan Xue

**Affiliations:** ^1^Department of Neuroscience, University of Florida College of Medicine and McKnight Brain InstituteGainesville, FL, USA

**Keywords:** Alzheimer’s disease, CNS immunosenescence, impaired neuronal protection, microglial cells, microglial senescence, neurodegeneration, neuropathological features, neuroprotection

## Abstract

This review is focused on discussing in some detail possible neuroprotective functions of microglial cells. We strive to explain how loss of these essential microglial functions might contribute toward the development of characteristic neuropathological features that characterize Alzheimer’s disease. The conceptual framework guiding our thinking is provided by the hypothesis that microglial senescence accounts for impaired neuronal protection and consequent neurodegeneration.

## Introduction

The role of CNS microglial cells in the development of Alzheimer’s disease(AD) has been the subject of considerable interest since McGeer’s initial description of reactive microglia in human AD brain in 1987 (McGeer et al., 1987). Most of the numerous studies that followed corroborated McGeer’s findings and collectively over the years they coalesced into the amyloid cascade-neuroinflammation hypothesis, which claims that neurodegenerative changes in AD (neurofibrillary degeneration) are the result of an uncontrolled, chronic intracerebral inflammatory reaction triggered by the accumulation/aggregation of amyloid-beta (Aβ) protein in plaques. Today, the neuroinflammation hypothesis is difficult to uphold given that clinical trials with anti-inflammatory drugs and strategies to remove Aβ from the brain have met with disappointing results and have yielded little in the way of effective treatments for humans. In this paper, we briefly describe our own vision of what may be the role of microglia in AD pathogenesis, which is different in that our primary focus is not on a single protein (Aβ) as the cause of AD, but instead relies on the incontrovertible fact that the incidence of sporadic AD is strongly correlated with aging. Our theory, which we call the microglial dysfunction hypothesis, states that neurodegeneration in AD is the result of an aging-related, gradually progressive breakdown of innate CNS immunity and loss of neuroprotection, i.e., microglial cell senescence. In contrast to normal aging this deterioration of innate CNS immunity appears to be accelerated in AD. The difference in perspective between the dysfunction hypothesis and the neuroinflammation theory is due in large part to our conviction that microglia are entirely beneficial cells, whose single-most important function is to provide neuronal protection at all times in the normal and injured CNS. In the following, we shall discuss succinctly several microglial neuroprotective functions and how senescent deterioration of these could contribute to development of neurodegenerative changes.

## The Brain’s Innate Immune System is Neuroprotective

Even though doubts about the very existence of microglia were expressed in textbooks until the early 1990s (Graeber, 2010), the notion that microglia are the key cellular elements comprising the brain’s innate immune system (Graeber and Streit, 1990; Streit and Kincaid-Colton, 1995) is now widely recognized. A common view that has been expressed many times is that together with peripheral macrophages, such as Kupffer cells of the liver, Langerhans cells of the epidermis, or alveolar macrophages in the lung, microglia are another member of the mononuclear phagocyte system, i.e., a tissue-specific macrophage. While there is little doubt regarding validity of a mononuclear lineage relationship, microglia in the CNS do not maintain a macrophage state constitutively or continuously. In the normal adult brain, microglia display a well-differentiated, dendritic morphology similar to that of other brain cells. They exhibit multiple, finely branched cytoplasmic processes with which they constantly explore the CNS microenvironment searching for disturbances that may require their quick response (Nimmerjahn et al., 2005; Tremblay et al., 2010). These ramified microglia are sensors of pathology (Kreutzberg, 1996; Stence et al., 2001; Petersen and Dailey, 2004; Davalos et al., 2005) and they do not exhibit macrophage morphology, nor do they express the typical macrophage marker, CD68 (recognized by ED1 antibody in the rat). Even activated microglia with hypertrophic morphology express the CD68 antigen only after having engaged in phagocytic activity (Graeber et al., 1998). It is therefore only under conditions of tissue/cell necrosis when debris must be phagocytized that microglia show expression of CD68 and can therefore be considered brain macrophages. The currently popular idea of differentiating functionally distinct macrophage phenotypes classified as M1 (cytotoxic), M2 (reparative), and a third, “deactivated” form might thus apply merely to those few microglial cells that at any given time are in a macrophage state during CNS injury or disease. To generalize and assume that the M1/M2 classification applies to the microglial population at large represents a rather indiscriminate extrapolation of *in vitro* studies performed with peripheral macrophages (Colton, 2009; Michelucci et al., 2009; Moon et al., 2011), and it could be misleading to assume that resting and activated microglia within the brain microenvironment function in the same manner as peripheral (professional) macrophages that have been cultured and manipulated *in vitro* with cytokines. There is currently no way of reliably identifying functionally distinct microglial phenotypes in brain tissue *in situ* and without this capability no real progress on functional involvement of putative M1 or M2 microglial subtypes in the cellular pathogenesis of AD can be made. In part because of this we are assuming that all microglia are potentially beneficial cells and our view of microglial involvement in AD is rooted firmly in that assumption. It is known that the CNS parenchyma represents a unique compartment that is segregated to a large extent from the rest of the body by the blood brain barrier (BBB), and that it represents an immunologically subdued environment (Ford et al., 1995; Perry et al., 1995; Carson et al., 1998; Hoek et al., 2000; Cardona et al., 2006), although not necessarily an “immunologically privileged site” (Galea et al., 2007). There are no constitutively active (professional) macrophages in the brain parenchyma under normal conditions. Our definition of a microglial cell is that of a cell type of the mononuclear cell lineage that has evolved to be highly adapted and specialized for residing within the unique CNS microenvironment. As such its immunological potential is limited largely to phagocytosis while its neuroprotective capabilities are maximized, and one might thus view microglia as a hybrid between a moderately immunocompetent mononuclear cell and a powerful neuroprotective glial cell.

We view the neuroprotective capabilities of microglia in the broadest possible meaning of the term, namely, anything that microglia do that is beneficial for and/or conducive toward proper neuronal functioning. One obvious beneficial function of microglia is simply their ability to transform into macrophages that can clear out debris and process waste when/if the need arises. These microglia-derived macrophages are not necessarily cytotoxic because much of the debris generated within the brain, for example after trauma or is chemia, is sterile (Chen and Nunez, 2010) and would thus not require elaboration of cytotoxins to kill microorganisms. Direct infection of the CNS with microorganisms is largely prevented by the natural barriers of skull, meninges, and BBB, but if these fail and when microorganisms do gain access to the parenchyma, they are likely to be eliminated quickly and efficiently by microglial cells and probably without too much collateral damage, as there are multiple mechanisms involved in post-infection neuropathology other than microglial neurotoxicity (Nau and Bruck, 2002; Mariani and Kielian, 2009; Ribes et al., 2011). One condition that deserves special mention is infection with the human immunodeficiency virus-1 (HIV-1), where microglial cells themselves are the target of the infectious agent (Michaels et al., 1988). It is therefore reasonable to assume that the functionality of HIV-infected microglia is compromised, although in ways that are not yet entirely understood. From our perspective, compromised microglial function includes primarily an impaired ability to provide neuroprotection, and it is perhaps because of this that patients with HIV/AIDS frequently suffer from the consequences of neurodegeneration, a.k.a. HIV-associated dementia (Anthony and Bell, 2008). It seems obvious that compromised innate immunity within the CNS due to HIV infection is what accounts at least in part for the high incidence of opportunistic CNS infections in HIV/AIDS patients, notably toxoplasmosis (Mariani and Kielian, 2009).

**Figure 1:**
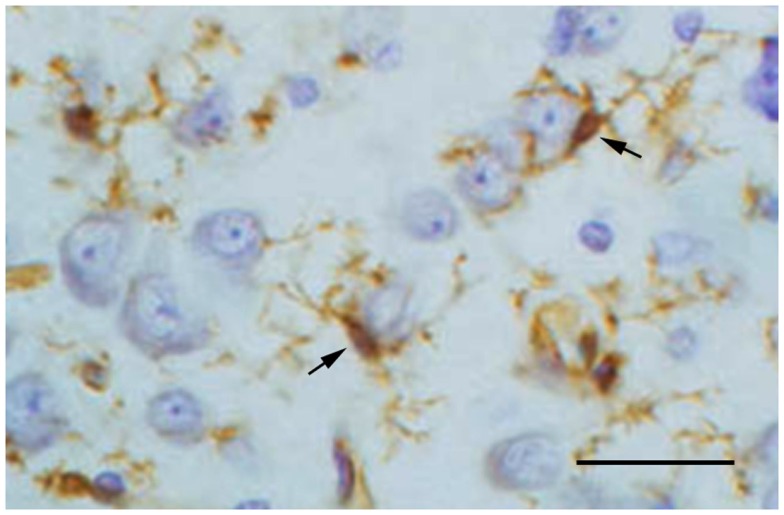
**Lectin staining of microglia (brown) in the normal cerebral cortex of a rabbit shows two microglial cells (arrows) extending their processes around cortical neurons**. This close spatial relationship suggests ongoing interactions between resting microglia and neurons. Neurons are stained with cresyl violet. Scale bar: 50 μm.

A second and very direct neuroprotective function provided by microglia is the production and secretion of neurotrophic factors, notably BDNF (Elkabes et al., 1996; Batchelor et al., 1999; Suzuki et al., 2001; Nakajima et al., 2002; Coull et al., 2005) and NGF (Mallat et al., 1989; Heese et al., 1997; Frade and Barde, 1998), but also others like TGF-β (Kiefer et al., 1993; Lehrmann et al., 1998), bFGF (Araujo and Cotman, 1992), and GDNF (Batchelor et al., 1999; Suzuki et al., 2001). Production of neurotrophins by microglia is usually increased after injury/during recovery when the cells become activated and neurons require more neurotrophic support than under normal conditions. However, even under normal conditions microglia are likely to sustain neuronal functioning during periods of high activity or sub-pathological stress when smaller doses of neurotrophic factors may be required. Although it is difficult to demonstrate directly that this type of specific microglial-neuronal interaction actually occurs, histological images showing some neurons covered tightly by microglial processes suggests that this could be the case (Figure 1). Different neuronal populations require different neurotrophic factors and quite possibly microglia have the ability to sense which factors and how much of them may be needed in any particular circumstance. Their ability to migrate and home in on neurons in need is essential for facilitating such targeted neuroprotection.

A third way in which microglia can be neuroprotective is by diverting noxious and potentially harmful substances away from neurons. It has long been known that astrocytes take up excess glutamate from the extracellular space under conditions of heightened neuronal activity in an effort to minimize glutamate-mediated excitotoxicity. During the past 10 years it has become clear that astrocytes are not alone in this effort and that activated microglial cells also participate in the removal of glutamate by upregulating primarily glutamate transporter-1 (GLT-1) *in vivo* following traumatic lesions as well as *in vitro* after stimulation with LPS, TNF-α, or neuronal conditioned medium (Lopez-Redondo et al., 2000; Nakajima et al., 2001, 2008; van Landeghem et al., 2001). Interestingly, glutamate uptake by microglia is coupled to enhanced glutathione synthesis by microglia, perhaps reflecting an effort by the cells to protect themselves from damage (Persson et al., 2006). These studies underscore the extensive crosstalk that takes place continuously between microglia and neurons. In addition to glutamate, a second substance with considerable and non-specific damage potential is free, redox-active iron, which may be present either as ferric (Fe^+3^) or ferrous (Fe^+2^) ions. We propose that the sequestration of free iron in the CNS by ferritin in microglia constitutes an important neuroprotective mechanism that becomes most relevant when there is a breach in the BBB and the possibility exists of free iron entering the brain parenchyma. The evidence for this mechanism of microglial neuroprotection is as follows: we and others have previously reported that many of the dystrophic (senescent) microglia in human brain are positive for the iron storage protein, ferritin (Simmons et al., 2007; Lopes et al., 2008), suggesting that the sequestration and concomitant accidental escape of free iron atoms can contribute to microglial senescence by increasing oxidative stress within these cells. This has raised the interesting and novel possibility that microglia rather than neurons may be primary victims of oxidative damage (Dringen, 2005; Lopes et al., 2008; Nakanishi and Wu, 2009), representing somewhat of a paradigm shift since in the past microglia have been seen primarily as a source of free radicals that endanger neuronal survival (Colton and Gilbert, 1987; Boje and Arora, 1992; Chao et al., 1992). It seems plausible then to think that microglia can protect neurons by taking the brunt of at least some oxidative stress and deflecting it away, which makes sense since microglia are relatively expendable and possess renewal capacity from within the CNS (mitosis) or from bone-marrow derived precursor cells. One might draw the analogy to the game of chess where pawns are sacrificed to save the more valuable pieces. Compatible with this idea of microglial self-sacrifice is evidence from animal experiments showing that activated microglia in the axotomized facial nucleus are negative for ferritin (unpublished) and that therefore ferritin expression in microglia is not necessarily linked to microglial activation. Importantly, transection of the facial nerve leaves the BBB undisturbed, but conversely, when a brain lesion does result in a breach of the BBB some microglia do become ferritin-positive and even dystrophic (Xue et al., 2010). Thus, neuroprotective microglia are like the HazMat Team in the CNS which will put itself in harm’s way to protect neurons.

A final aspect of microglial neuroprotection within the scope of this discussion concerns their emerging role in the regulation of the plasticity of neuronal circuits, that is, their involvement in the pruning/elimination and maintenance of synaptic connections. This aspect of the microglial functional repertoire was recognized for the first time more than 40 years ago by Blinzinger and Kreutzberg (1968), yet it remained largely unexplored until quite recently when a number of laboratories started to reexamine and delve deeper into this phenomenon. It now appears that microglial regulation of neuronal connectivity is important during development, as well as in the normal and injured adult brain (Wake et al., 2009; Tremblay et al., 2010; Paolicelli et al., 2011). The stripping of synapses from axotomized motoneurons (Blinzinger and Kreutzberg, 1968) perhaps best illustrates a direct neuroprotective effect of such cellular action in that displacement of synapses from the surface of injured motoneurons may prevent afferent excitation, which is hardly needed at a time when motoneurons are working to regenerate their severed axons (Streit, 1993). In fact, tight microglial ensheathment of injured motoneurons instantaneously fulfills multiple goals of neuroprotection: while preventing unnecessary excitation through synaptic displacement, it also puts microglia into very close proximity to the neuronal cell soma to facilitate targeted delivery of neurotrophic factors and, in addition, it perfectly prepositions microglia for rapid phagocytosis should a neuron fail to survive as a result of having been axotomized. Elimination of synapses during development (Paolicelli et al., 2011) or during altered sensory processing (Tremblay et al., 2010) is neuroprotective insofar as it facilitates the proper formation and rearrangement of synaptic connections and thus optimizes neuronal functioning.

## Neuroinflammation and the Significance of Microglial Activation in AD

**Figure 2:**
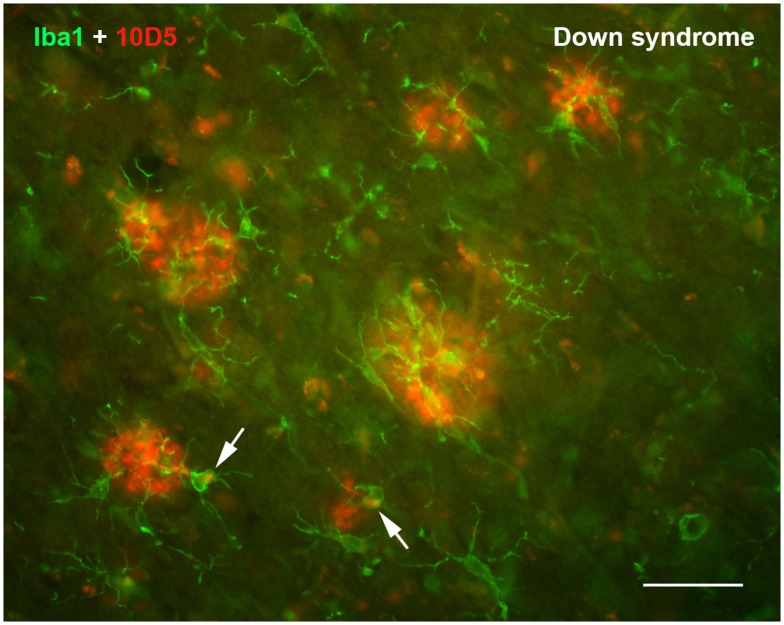
**Double immunofluorescent staining for microglia (Iba1) and Aβ protein (10D5) in the cerebral cortex of a human with Down syndrome reveals apparently normal, non-activated microglia in and around Aβ deposits**. Microglia are ramified and show no evidence of having internalized any Aβ protein. Two microglial cells show presence of intracellular, autofluorescent lipofuscin (arrows). Scale bar: 50 μm.

Countless studies in laboratory animals involving experimental CNS lesions have shown that microglia become activated rapidly in response to neuronal injury (Kreutzberg, 1996; Kettenmann et al., 2011). This prompt cellular reaction to CNS tissue injury (glial activation), which can occur within seconds after injury (Nimmerjahn et al., 2005), constitutes acute neuroinflammation. The primary purpose of such acute inflammation, being limited in its range to the immediate vicinity of the lesion, is to initiate wound healing and to restore homeostasis as soon as possible, and microglial activation subsides as healing occurs. Excess microglia generated during the activation response *via* mitosis of resident cells undergo programmed cell death which reduces cell numbers back to baseline (Gehrmann and Banati, 1995; Jones et al., 1997; Conde and Streit, 2006), constituting a form of physiological cell death perhaps similar to what happens in normal ontogenetic development. One seemingly trivial insight to be gained from these lesion studies is that they clearly establish the cause-and-effect relationship between injury and subsequent microglial activation underscoring the fundamental definition of inflammation, i.e., the cellular response to injury. In the context of AD and the amyloid cascade-neuroinflammation theory a cause-and-effect relationship is anything but trivial. Although deposition of the Aβ protein is seen by many as a trigger for microglial activation in AD and has thus given rise to a large body of literature resulting from sundry experimental approaches, there is no consensus to date whether or not Aβ actually stimulates microglial activation. Case in point, we and others have observed that some human brains with substantial Aβ loads reveal a notable lack of microglial activation (Itagaki et al., 1989; Ohgami et al., 1991; Streit et al., 2009; Figure 2). Similarly, the supposition that microglial cells are involved in the clearance of Aβ has not been proven. Generally speaking, studies performed *in vitro* with cultured microglia provide the only convincing evidence that microglia can phagocytize Aβ protein, but these stand in stark contrast to most studies in Aβ-overexpressing animal models (which do not) and certainly to studies in human brain which reveal a lack of Aβ phagocytosis by microglia. Thus, the relationship between Aβ, microglial activation, and/or neuroinflammation remains enigmatic and perplexing even after more than 20 years of intense research. If one also considers the alleged causal connection between amyloid deposits and neurofibrillary degeneration the picture gets blurred even more as this connection seems to grow weaker and evidence is mounting against rather than in favor of it.

Yet another issue that complicates our understanding of neuroinflammation in AD is found in the fact that primary infections outside of the CNS influence the state of microglial cells. In particular, sepsis, which is more commonly found in elderly patients due to compromised immune function, will induce microglial activation within the brain (Lemstra et al., 2007; Streit et al., 2009). It is likely that there are other types of systemic pathology that may do so as well (Mattiace et al., 1990). Nearly all prior studies that have reported microglial activation and/or upregulation of inflammatory cytokines in the CNS of AD subjects have not made a distinction between cases that were free of peripheral infectious disease and those that were not, and it is therefore quite possible that neuroinflammatory changes reported could have been the result of peripheral diseases. Studies are underway in our laboratory to further investigate this possibility.

## Microglial Senescence and Neurodegeneration – Connecting the Dots

The discovery of dystrophic microglia in human brain represents a critical step in conceiving the microglial dysfunction hypothesis because it raises the possibility that microglia are subject to senescence and degeneration (Streit et al., 2004). Dystrophic microglia were first identified in the aged human brain as cells displaying abnormal morphological features, such as shortened, gnarled, beaded, or fragmented cytoplasmic processes, as well as loss of fine ramifications and formation of spheroidal swellings. Because they are present in greater numbers in aged vs. young humans microglial dystrophy is thought to reflect degenerative changes related to cell senescence. Our subsequent demonstration of a close spatial and temporal relationship between neurofibrillary degeneration (tau pathology) and microglial dystrophy in subjects with either AD or Down syndrome has provided another crucial piece in the puzzle consolidating the dysfunction theory by linking microglial senescence and neurodegeneration (Streit et al., 2009; Xue and Streit, 2011). Additional support for this link is derived also from the well-known fact that aged rodents typically do not develop neurofibrillary degeneration, and that microglial degeneration has been undetectable in uninjured rodent brain (Streit and Xue, 2010). Clearly, the next major step in advancing this line of thinking would be to induce microglial dystrophy experimentally and determine if it is accompanied by neurodegenerative changes. Current efforts in our laboratory are directed toward that goal.

**Figure 3:**
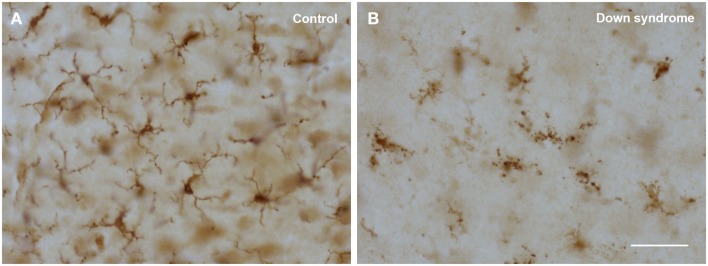
**Comparison of normal (ramified) and degenerating (dystrophic) microglia using Iba1 immunostaining in human cerebral cortex**. **(A)** 22-year-old male non-demented subject reveals cells with normal morphology; **(B)** 48-year-old female subject with Down syndrome shows cells displaying obvious cytoplasmic fragmentation. Scale bar: 50 μm.

The morphological abnormalities that characterize dystrophic microglia have been described in detail before (Streit et al., 2004, 2009; Xue and Streit, 2011), suffice it to say here that the most advanced and striking change involves fragmentation of the cells’ cytoplasm, which is termed cytorrhexis (Figure 3). Cytorrhexis reflects obvious degeneration of cell structure and it appears to be the end result of a progression from beading of processes to subsequent fragmentation not unlike what has been observed during axonal degeneration (Kerschensteiner et al., 2005; Coleman and Freeman, 2010). We view microglial cytorrhexis as a form of accidental cell death with as of yet unknown causes, although much points toward undue oxidative stress as a likely etiologic factor. Our prior work has shown that cytorrhexis does not involve detectable nuclear fragmentation further supporting the idea of an accidental rather than an apoptotic mechanism (Fendrick et al., 2007).

With regard to the aforementioned neuroprotective roles of microglia, there are a number of interesting points to consider in terms of how deterioration of these neuroprotective functions could be critically important in the development of aging-related neurodegenerative diseases. Phagocytosis of debris by microglia is an essential cellular activity that ensures maintenance of a clean, debris-free brain parenchyma, and is conducive toward facilitating optimal interneuronal electrochemical signaling. A substantial decline in microglial phagocytosis due to cell senescence and/or degeneration would almost certainly contribute to NDD development (Neumann et al., 2009). In addition, impaired phagocytosis could be a factor contributing to reduced clearance of amyloid deposits, a possibility that has been raised in previous reports (Fiala et al., 2005; Streit et al., 2008; Njie et al., 2012). Areas of the human brain showing advanced tau pathology are characterized by widespread presence of cytorrhectic microglia but also reveal a conspicuous absence of phagocytic activity. This very likely reflects the fact that fragmented microglia cells no longer are capable of performing phagocytosis, which explains why areas of tau pathology are littered with uncleared microglial debris. Although not yet shown, it would hardly come as a surprise to find out that the ability of cytorrhectic microglia to produce sufficient amounts of neurotrophic factors is much reduced. The possibility also exists that dying microglial cells could elaborate toxic factors, which might be a way to reconcile the dysfunction and neuroinflammation ideas.

For quite some time now the presence of increased brain iron and/or a disturbance in iron metabolism has been seen as a major factor contributing directly to free radical mediated neuronal degeneration (Jellinger et al., 1990; Dexter et al., 1991; Youdim and Riederer, 1993; Lynch et al., 2000; Berg et al., 2001; Zecca et al., 2004; Smith et al., 2010). Here again, microglial degeneration offers an opportunity to deepen our understanding of the importance of brain iron in the pathogenesis of neurodegenerative diseases. As levels of brain iron increase with aging, perhaps due in part to presence of microbleeds, the burden for resident microglial cells in sequestering free iron through ferritin expression becomes progressively higher. At the same time the risk for microglia to develop degenerative changes through iron-mediated oxidative stress is heightened, thus resulting in an ever increasing number of dystrophic and dysfunctional microglial cells with impaired ability to provide neuroprotection. Observations in humans showing that a large proportion of ferritin-positive microglia are dystrophic and that these dystrophic cells accumulate in advanced lesions (senile plaques) substantiate this line of thinking (Kaneko et al., 1989; Lopes et al., 2008; Xue and Streit, 2011).

As mentioned, there is an increasing number of recent studies that suggest significant involvement of microglia in synaptic plasticity (Tremblay et al., 2011). If microglia are indeed the electricians of the brain and important for maintaining synaptic integrity of neuronal circuits (Graeber, 2010), then their deterioration in the AD brain could certainly play a direct role in the loss of synapses which represents a hallmark feature of the disease (DeKosky and Scheff, 1990; Terry et al., 1991; Lassmann et al., 1993). Naturally, if the dysfunction hypothesis is correct and microglial degeneration contributes to neurodegeneration, the loss of synapses secondary to neuronal degeneration would also fit into this scenario. Some studies have suggested a role for microglia in synapse elimination as well as in synaptogenesis during CNS development (Bessis et al., 2007), and these functions are likely to be performed much more effectively by young microglial cells in the developing brain than by senescent ones in the aged CNS.

## Conclusion

As the brain’s innate immune system one might be inclined to think of microglia primarily as immunological defenders that fight invading microorganisms. However, in so doing one underestimates their importance as supportive and neuroprotective glial cells essential for helping to maintain neuronal functioning in the normal CNS and especially their crucial involvement in CNS repair and regeneration during injury and disease. We believe that microglial neuroprotection constitutes a most important aspect of the cells’ biological significance because it defines a single common denominator that contributes to improved understanding of CNS development, normal adult brain function, as well mechanisms of injury, disease, and repair. Specifically, with regard to aging-related neurodegenerative diseases, which represent one of the greatest challenges for biomedical science in this day and age, we think that the concept of CNS immunosenescence has considerable potential for advancing progress in terms of new and improved approaches toward treatment and prevention.

## Conflict of Interest Statement

The authors declare that the research was conducted in the absence of any commercial or financial relationships that could be construed as a potential conflict of interest.
